# Synthesis of Urea-Formaldehyde Microcapsule Containing Fluororesin and Its Effect on Performances of Waterborne Coatings on Wood Surface

**DOI:** 10.3390/polym13111674

**Published:** 2021-05-21

**Authors:** Xiaoxing Yan, Yan Han, Taiyu Yin

**Affiliations:** 1 Co-Innovation Center of Efficient Processing and Utilization of Forest Resources, Nanjing Forestry University, Nanjing 210037, China; 2 College of Furnishings and Industrial Design, Nanjing Forestry University, Nanjing 210037, China; hanyan@njfu.edu.cn (Y.H.); yintaiyu@njfu.edu.cn (T.Y.)

**Keywords:** microcapsule, urea-formaldehyde resin, fluororesin, coating performance

## Abstract

In order to self-repair the cracks of waterborne coatings on Basswood at room temperature, with fluororesin and waterborne coatings embedded in the shell structure of urea formaldehyde (UF) resin, the microcapsules were fabricated via in-situ polymerization, and the effect of microcapsules on the chroma, gloss, mechanics and repair effect for waterborne coatings on wood was discussed. The results indicated that the coating effect was the most significant when the ratio value of the core materials to the shell material of microcapsules in mass was 0.75, and the agglomeration of particles was the least and the surface was the smoothest when the content of microcapsules was 1.0%. It was negative between the gloss of the film and microcapsule content. The ratio value of the core materials to the shell material in mass and the amount of microcapsules had great influence on the film hardness and adhesion, but had little effect on the impact resistance. When the ratio value of the core materials to the shell material of microcapsules in mass was 0.65 and the addition amount was 4.0–10.0%, the aging resistance of the film was improved most significantly. When the ratio value of the core materials to the shell material of microcapsules in mass was 0.65 and the addition amount was 7.0%, the overall properties of topcoat film on Basswood board was the most significant. It is for the application of fluororesin microcapsules possessing self-repairing effect in waterborne coating on Basswood board that a technical groundwork is provided by this study.

## 1. Introduction

The film of waterborne coatings has the characteristics of stable property, high density, easy repairability and excellent anticorrosion performance [1,2,3]. It does not contain toxic organic solvents such as benzene, aldehydes and halogenated hydrocarbons, heavy metal compounds such as lead and chromium, and formaldehyde, which is non-toxic and environmentally friendly [4,5]. As a safe and pollution-free environmental protection coating, it basically contains no volatile substances and has no unfriendly odor [6,7]. With the increasingly strict control of indoor VOCs standards [8], the traditional solvent-based coatings are gradually replaced, and the application of natural coatings and waterborne coatings is more and more extensive [9]. However, it is found that waterborne coatings have some disadvantages such as poor water resistance and mechanical properties [10].

As a kind of common material, Basswood is generally used for blockboard, wooden strip and wooden handicrafts [11,12,13]. Basswood has light weight, soft texture, fine and uniform texture, good mechanical processing performance, low aging degree, fast drying speed, but large shrinkage rate during drying. This obvious wet swelling and shrinkage is not conducive to the processing and use of Basswood. Under the interference of external environmental factors, the waterborne coating on the surface of Basswood is very easily affected to produce subtle deformation in the use process, which makes the coating produce microcracks [14]. If the microcracks are not repaired, they will develop into cracks, which make the performance of the coating degrade rapidly. The service life of coatings will be shortened because of these microcracks [15]. Artificial repairing on coatings is prone to uncontrollable thickness, and the repaired parts are prone to causing defects again or even peeling due to the presence of microcracks in the internal coatings [16]. Inspired by the mechanism of biological self-healing, the self-healing technology has been applied in the field of coatings, endowing polymer materials with “life”. The self-healing makes the polymer materials repair themselves like living bodies when they are damaged, to have the function of “sensing”. Thus, the hidden danger caused by the microdamage which cannot be detected by the naked eye in time is eliminated, and the service life of the material is prolonged [17].

A microcapsule is a kind of micro container which is made of polymer material as wall material to cover the repair agent, which can protect the core material from environmental impact [18,19,20]. The wall of the microcapsule will be destroyed by external force when micro cracks occur in the coating [21]. The core material containing a repair agent can repair the cracks [22]. The application of microcapsule technology in the coating on wood surface can repair the micro-cracks in time at the early stage of microcrack formation that cannot be perceived by the naked eye [23], avoid causing micro cracks or even breaks to improve the service life of the coating film and prolonging the service life of wood. Parsaee et al. [24] used epoxy resin and urea-formaldehyde/polyurethane via in-situ polymerization. It was fabricated successfully that the microcapsules would have good self-healing ability and corrosion resistance on the surface of low carbon steel due to the contained epoxy resin. Li et al. [25] reported the synthesis of graphene oxide microcapsules (GOMC) containing flaxseed oil as healing agent via self-assembly technique. GOMC was embedded in waterborne polyurethane matrix, which made it easy to fabricate self-repairing composite coatings on the surface of hot-dip galvanized steel. Najjar et al. [26] fabricated silica-coated isophoronediisocyanate (IPDI) in aqueous polyurethane coatings. It was found that the microcapsules containing IPDI not only enabled the coating to have self-healing properties, but also improved the thermal stability of the coating. Schreiner et al. [27] prepared microcapsules with good self-healing ability by using thermosetting resins (urea-formaldehyde resin and melamine-formaldehyde) as substrates. Additionally, the self-healing process of the microcapsules needs to be carried out at a higher temperature. These microcapsules are self-repairing for waterborne coating. However, there are few studies on microcapsules which can solidify at room temperature to achieve the self-healing effect and microcapsules suitable for the surface coating on Basswood.

Fluororesin materials possess high thermal stability, weatherability, acid-base resistance and solution resistance [28], and are widely used in aerospace and special protective coatings and other fields [29]. At present, the commercial waterborne fluoro coatings are divided into two categories: baking film-forming coatings and room temperature film-forming coatings. Among them, fluoroolefin/vinyl ether waterborne coatings and vinylidene fluoride copolymer waterborne coatings can be formed at room temperature. Because wood materials are not resistant to high-temperature baking, the application of fluororesin film-forming at ambient temperature in microcapsule technology has certain research significance for waterborne self-repairing coatings on wood surface.

Yan et al. have preliminarily explored the application of microcapsule self-healing technique in coatings, and applied microcapsules in waterborne coatings for better comprehensive performance [14,30]. Based on this foundation, the microcapsules with several different ratios of the core materials to the shell material in mass were fabricated using UF resin as shell material, the integrated body of vinylidene fluoride resin and waterborne coating as core material. The effects on chromatic aberration, gloss and hardness analysis of the waterborne coatings were discussed by changing the value of addition and adopting two-bottom and two-side coating process. The aging resistance and self-healing performance of the coatings was preliminarily discussed for better. In this paper, it was realized that the self-healing effect of waterborne coatings with fluororesin microcapsules at room temperature, and the effects on the optical, mechanical and aging properties of waterborne coatings on Basswood board were explored, which provided technical reference for the self-healing ability of waterborne coatings on the surface of wood products.

## 2. Materials and Methods

### 2.1. Experimental Materials

Vinylidene fluoride FR904 (PVDF, resin, M_w_: 64.03 g/mol, CAS No.: 24937-79-9, Industrial, purity ≥ 99.9%) was offered by Zhanyang Polymer Materials Co., Ltd., Dongguan, China. N-Methyl pyrrolidone (NMP, M_w_: 99.13 g/mol, CAS No.: 872-50-4 Industrial, purity ≥ 99.9%) was offered by Zhanyang Polymer Materials Co., Ltd., Dongguan, China. Formaldehyde solution (M_w_: 30.03 g/mol, CAS No.: 50-00-0, 37%), urea (M_w_: 60.06 g/mol, CAS No.: 57-13-6) and triethanolamine (TEOA, M_w_: 149.19 g/mol, CAS No.: 102-71-6) were offered by Yatai United Chemical Co., Ltd., Wuxi, China, which are all analytically pure. Sodium dodecyl benzene sulfonate (SDBS, M_w_: 348.48 g/mol, CAS No.: 25155-30-0) was offered by Zhiyuan Chemical Reagent Co., Ltd., Tianjin, China, which is analytically pure. The waterborne coating is constituted of aqueous acrylic copolymer dispersion (90.0%), duller (2.0%), additive (2.0%) and water (6.0%). Basswood boards (65 mm × 100 mm × 5 mm) were offered by Yihua Life Technology Co., Ltd., Shantou, China.

### 2.2. Experimental Methods

The experiment was divided into two steps. In the first step, the microcapsules are prepared. In the second step, the fabricated microcapsules are added to waterborne paint and the coating properties are tested. Three kinds of microcapsules were prepared according to different ratios of core material to wall material (Table 1).

Fabrication of wall prepolymer: at a mass ratio of 1:1.35, the 20.00 g urea was added in the beaker and so was the 27.00 g formaldehyde solution. Both were stirred and mixed uniformly. The triethanolamine was added slowly to control the pH value of the mixed solution at about 8.0 to 9.0. The prepared solution was stirred constantly for 1.5 h in the magnetic stirrer at 70 °C. The obtained UF prepolymer solution was slightly viscous and transparent, and then the wall prepolymer solution was taken out and chilled to room temperature for standby.

Fabrication of core material: the polyvinylidene fluoride resin powder and NMP solution were weighed at a ratio of 1:8 and mixed evenly, the waterborne coating was weighed at 1:9 ratio of polyvinylidene fluoride resin powder and added to the mixed polyvinylidene fluoride solution. As a kind of surfactant (emulsifier), the mixture was added with 150 g aqueous solution containing 1.0% sodium dodecyl benzene sulfonate. At 60 °C, the mixed solution was stirred uniformly in a magnetic stirrer at a speed of 900 r/min to fabricate a slightly viscous transparent core emulsion.

Microencapsulation: under the rotational speed of 900 r/min, as the wall material of microcapsules, the chilled UF resin solution was dropwise instilled to the emulsion with core materials. The pH value of the emulsion was controlled to 2.5–3.0 via slow dripping of the citric acid crystal. With the temperature slowly controlled to rise to about 70 °C, the solution reacted through continued stirring for 3 h. The obtained product was cooled at room temperature and left to stand for 7 d. The microcapsule white powder was obtained by filtration, washing with ethanol and deionized water for several times, and then drying at 30 °C for 24 h.

The microcapsules were mixed into the waterborne coatings: with the waterborne coatings as the paint base, seven parts of 2.0 g waterborne topcoats with the microcapsule contents of 1.0%, 4.0%, 7.0%, 10.0%, 13.0%, 16.0% and 20.0% were prepared, respectively. The coating process was two bottoms and two sides, in which the primer was varnish and the top coat was mixed with fluororesin microcapsules. Each coating was evenly coated on the surface of Basswood board and solidified at room temperature for 3 h.

### 2.3. Testing and Characterization

The chromaticity values of the coatings film was measured by SEGT-J portable chromatic aberration instrument (Shenzhen Threenh Technology Co., Ltd., Shenzhen, China) and the chromatic aberration value was calculated. The gloss of coatings film was examined by GB/T 4893.6-2013 “Physical and chemical properties test of furniture surface paint film Part 6 Gloss determination method” [31]. The adhesion of coatings film was examined by GB/T 4893.4-2013 “Physicochemical performance test of furniture surface paint film Part 4 determination of adhesion cross-cutting method” [32]. The shock resistance of paint film was gauged by GB/T 4893.9-2013 “Physical and chemical properties test of furniture surface paint film Part 9 Determination of impact resistance” [33]. The microscopic structure and chemical constitution of the coating film were gauged by environmental scanning electron microscopy (SEM) (FEI Company, Hillsboro, OR, USA) and VERTEX 80V infrared spectrometer (Germany BRUKER Co., Ltd., Karlsruhe, Germany). The thermal aging resistance of the coating film was gauged by a high temperature drying experiment in an electrothermal constant temperature blast oven (Tianjin Taist Instrument Co., Ltd., Tianjin, China). Basswood boards were baked at 120 °C for 2 h, 140 °C for 2 h, and 160 °C for 2 h in the oven. The aging resistance was assessed by observing and comparing the changes of gloss reduction and chromatic aberration of the coatings. The ultraviolet (UV) aging experiment was carried out in UV aging test chamber (Dongguan Jiedong Experimental Equipment Co., Ltd., Dongguan, China). The mixture composed of paraffin and colophony was coated on the back and sides of the Basswood pattern to obtain a coating measuring 10.0 cm by 5.0 cm. Subsequently, the coatings were processed for 240 h in the UV aging test chamber. The effect of self-healing was judged by observing the change of scratch width under the microscope after scratching the coating with a scalpel blade. All the tests were repeated four times, and the error was within 5.0%.

## 3. Results and Discussion

### 3.1. Effect of Microcapsules on Optical Properties of Waterborne Paint Film on Basswood Surface

#### 3.1.1. Chromatic Aberration Analysis of Paint Film

The chromatic aberration value is used to represent the uniformity of the surface color of the material, expressed in ΔE. In the lab color model, L represents illumination, a* represents the range of colors from red to green, and b* represents the range of colors from yellow to blue. The h* stands for hue and c* for chroma. ΔL (the difference of illumination) = L_1_ − L_2_, Δa* (the difference from red to green) = a_1_ − a_2_, Δb* (the difference from yellow to blue) = b_1_ − b_2_. The chromatic aberration value is computed in accordance with formula (1):

ΔE = [(ΔL)^2^ + (Δa*)^2^ + (Δb*)^2^]^1/2^ (1)

**Figure 1:**
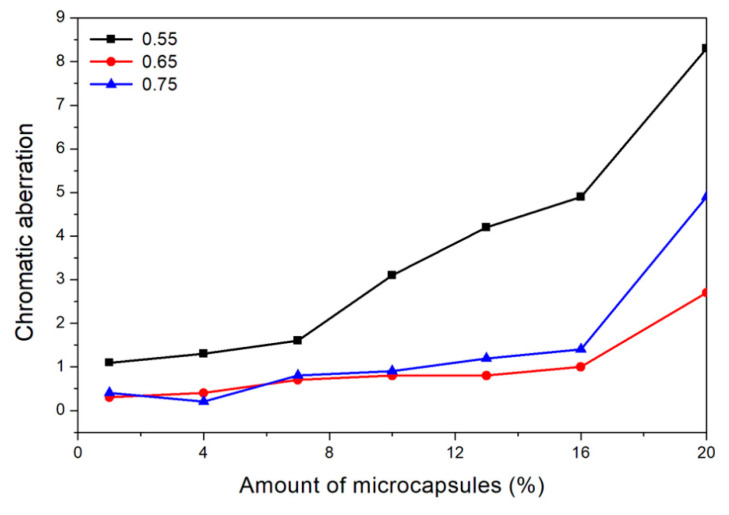
Effect of microcapsules on chromatic aberration of paint film.

As shown in Figure 1, the chromatic aberration of waterborne coatings on the Basswood board increases with the amount of microcapsules added. When the amount of microcapsule is 1.0%, the chromatic aberration of waterborne coating on wood surface is the smallest and the least affected by microcapsules. When the amount of microcapsule is 20.0%, the chromatic aberration of waterborne coating on Basswood board is the largest and the most affected by microcapsules. The range of fluctuation is not large when the addition amount is between 1.0% and 10.0%, and the overall growth trend of chromatic aberration value is relatively flat, but the range of chromatic aberration value of waterborne coating on wood surface increases sharply when the addition amount is between 10.0% and 20.0%, and the growth trend is more obvious. For these three kinds of microcapsules with different ratios of the core materials to the shell material in mass, the chromatic aberration of the waterborne coating on the Basswood board becomes larger with the increase in the value of ratio of core materials to shell material in mass at the same addition amount. Therefore, the addition amount of microcapsules was positively correlated with the chromatic aberration of waterborne coating on Basswood board, and the mass ratio of the core materials to the shell material of microcapsules was also positively correlated with the chromatic aberration of waterborne coating on wood surface. In conclusion, after the content of microcapsules in waterborne acrylic acid exceeds 10.0%, the higher the content, the more uneven the color of the wood surface covered by the coating. It greatly affects the decoration effect and aesthetics.

#### 3.1.2. Gloss Analysis of Paint Film

**Figure 2:**
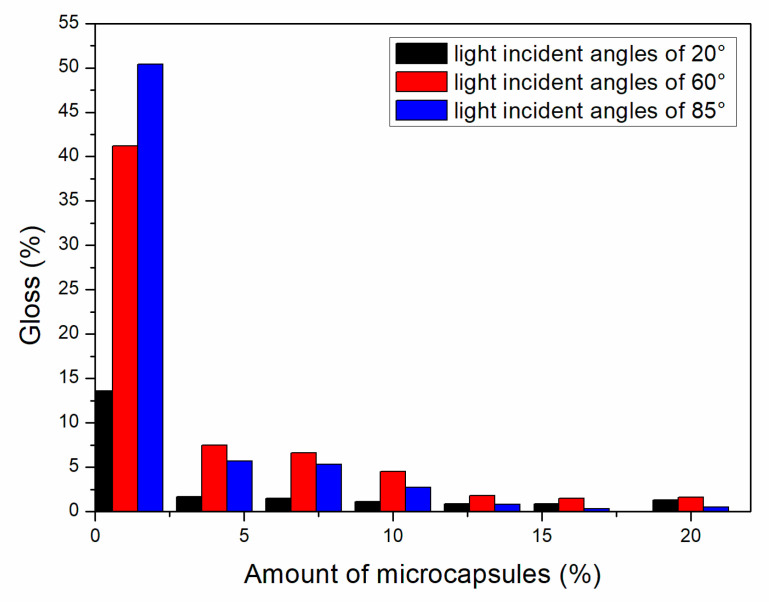
Effect of microcapsule content with the ratio of core materials to shell material in mass of 0.55 on gloss of coating film.

**Figure 3:**
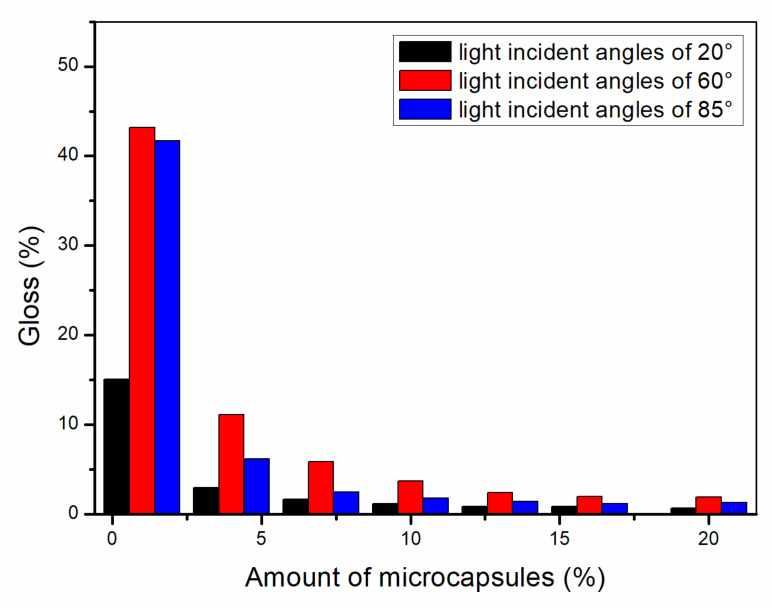
Effect of microcapsule content with the ratio of core materials to shell material in mass of 0.65 on gloss of coating film.

**Figure 4:**
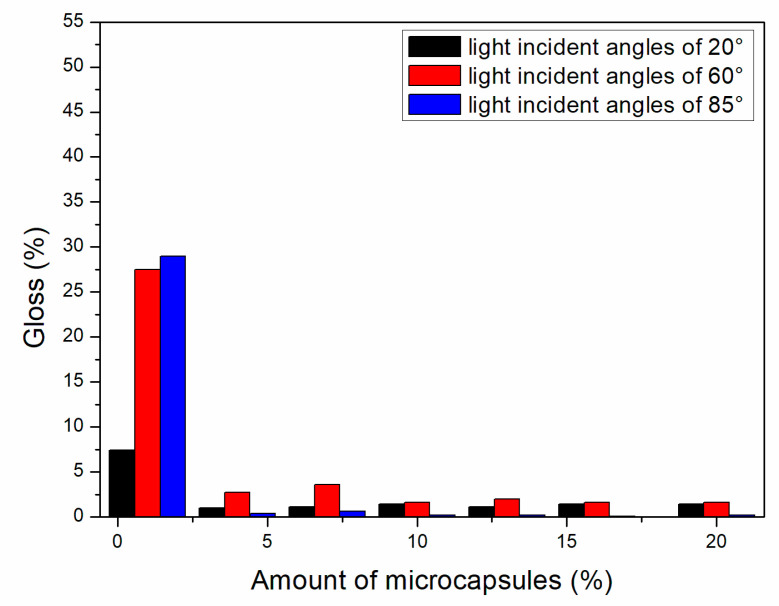
Effect of microcapsule content with the ratio of core materials to shell material in mass of 0.75 on gloss of coating film.

As shown in Figure 2, Figure 3 and Figure 4, the gloss of the coating was tested with three light incident angles at 20°, 60° and 85°, respectively. The amount of microcapsules had a great influence on the gloss of the coatings on Basswood surface. There was a negative correlation between the gloss and the amount of microcapsules. The greater the addition of microcapsules, the lower the gloss of the coating on the wood surface. From a horizontal perspective, the gloss of the coating decreased dramatically when the amount of microcapsules ranged from 1.0% to 4.0%. When the amount of microcapsules ranged from 4.0% to 20.0%, the downward trend slowed down although it was still declining. In addition, when the incident angle of light was the same, the coating gloss grows negatively as the amount of addition became larger. When the content of microcapsules is the same, the samples with two kinds of microcapsules with ratios of the core materials to the shell material in mass of 0.55 and 0.65 had relatively less effect on the coating gloss, while the sample with microcapsules with the ratio of core materials to the shell material of 0.75 had a greater influence on the coating gloss. Compared with 0.75, the fluctuation range of the coating gloss of the microcapsules with ratios of the core materials to the shell material in mass of 0.55 and 0.65 was smaller, and the decline trend was also slower. The gloss of coating on Basswood surface was negatively affected by the amount of microcapsules added. The coatings with mass ratios of the core materials to the shell material of 0.55 and 0.65 had better gloss. When the amount of microcapsules was 1.0%, the gloss of coating on Basswood surface was the most significant. When the amount of microcapsule was 20.0%, the gloss of waterborne coating on wood surface was the worst.

### 3.2. Effect of Microcapsules on Mechanical Performances of Waterborne Paint Film on Basswood Surface

#### 3.2.1. Analysis of Impact Resistance of Paint Film

According to Table 2, the impact strength of coating on Basswood surface was also affected by microcapsules. When the ratio of the core materials to the shell material in mass was 0.55, the impact strength of the waterborne coating with the addition of 13.0% microcapsule was the most significant. When the ratio of the core materials to the shell material in mass was 0.65, the impact strength of the waterborne coating with the addition of 7.0% microcapsule was the best. When the ratio of the core materials to the shell material in mass was 0.75, the impact strength of the waterborne coating with the addition of 7.0–16.0% microcapsule was the best. The microcapsule content enhanced the shock resistance of the paint film. The increase in microcapsule content is conducive to the stress transfer between the particles and the matrix, so that the load capacity becomes stronger.

#### 3.2.2. Hardness Analysis of Paint Film

In Table 3, the amount of microcapsules and the hardness of the coating on the Basswood surface were related although the effect was not significant. There was a positive correlation. The greater the amount of microcapsule added, the greater the hardness of the coating on the Basswood surface. In addition, it was negative between the ratio of the core materials to the shell material of the microcapsules in mass and the coating hardness on Basswood board. The greater the ratio of the core materials to the shell material of the microcapsules in mass, the smaller the waterborne coating hardness on Basswood surface. This may be due to the larger amount of the core material resulting in a reduction in the hardness of coatings.

#### 3.2.3. Adhesion Analysis of Paint Film

The evaluation standard of film adhesion is divided into six grades. Grade 0 is the best and grade 5 is the worst. Because the coating has good adhesion, the amount of fluororesin microcapsules has little effect on the adhesion of coating on Basswood surface. The fluororesin microcapsules with ratios of the core materials to the shell material in mass of 0.55 and 0.65 have less effect on the adhesion of coatings on Basswood surface. The film adhesion with ratio of the core materials to the shell material in mass of 0.75 decreased in turn from grade 0 to 3, which was due to the agglomeration effect of microcapsule content on the film adhesion according to Table 4. Whether 1.0% to 7.0% microcapsules were added, the adhesion of the coating was basically in the optimal state.

### 3.3. Microstructure Analysis

**Figure 5:**
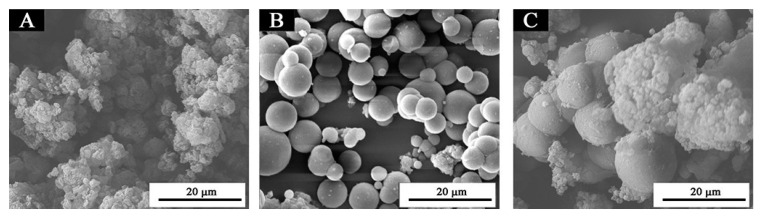
SEM images of microcapsules with different ratios of core materials to shell material in mass. (**A**) the ratio of core materials to shell material in mass of 0.55, (**B**) the ratio of core materials to shell material in mass of 0.65, and (**C**) the ratio of core materials to shell material in mass of 0.75.

**Figure 6:**
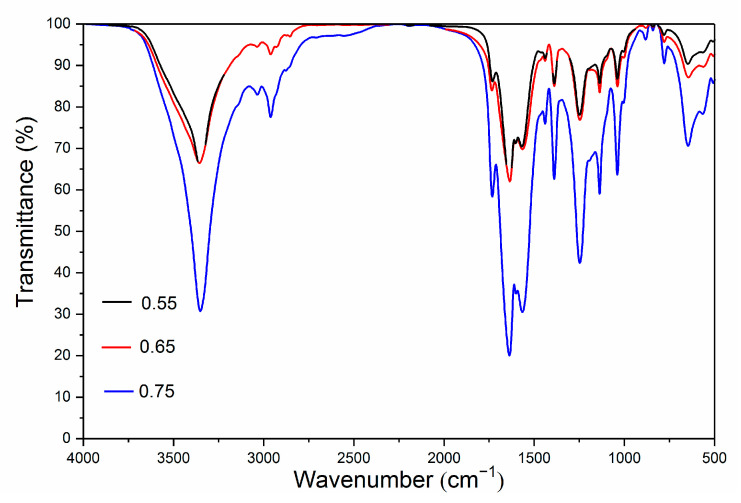
Infrared spectrum of microcapsules with different ratios of core materials to shell material in mass.

From Figure 5, the samples with the ratio of core materials to shell material in mass of 0.55 have more amorphous substances. Samples with the ratio of core materials to shell material in mass of 0.65 are basically spherical with small particle size. Samples with the ratio of core materials to shell material in mass of 0.75 are also spherical, but the size is uneven, and the surface is smooth. From the results of scanning electron microscopy analysis, the samples with different ratios of core materials to shell material in mass of 0.65 and 0.75 were initially presumed to be successfully coated by microcapsules. Three kinds of microcapsule powders with different ratios were tested by infrared spectroscopy, as shown in Figure 6. There is a high absorption peak at 3438 cm^−1^, which is mainly attributed to N-H stretching vibration of the amino group. The absorption peak at 2918 cm^−1^ is caused by C-H stretching vibration of -CH_2_, and there is also a strong absorption peak at 1632 cm^−1^, which is caused by N-H bending vibration of the amino group. There are strong absorption peaks at 1550 cm^−1^ and 1348 cm^−1^, which are mainly caused by the stretching vibration of C=N double bond and C-N single bond. The characteristic absorption peaks of the above infrared spectra correspond to the chemical bonds of the UF resin, which indicates that the composition of the microcapsules contains urea-formaldehyde resin. At 1385 cm^−1^, 1148 cm^−1^, 977 cm^−1^, 768 cm^−1^, 610 cm^−1^, 535 cm^−1^, the characteristic absorption peaks of the infrared spectrum of polyvinylidene fluoride appeared, which indicates that the microcapsules contained polyvinylidene fluoride. The characteristic absorption peaks of infrared spectra of water-borne acrylic acid were shown at 1730 cm^−1^, 1250 cm^−1^, 1160 cm^−1^ and 1050 cm^−1^, which represents that the microcapsules contained water-borne acrylic acid. The composition of the wall material and core material of the microcapsule is reflected by infrared spectroscopy, which shows that the encapsulation of the microcapsule is successful.

**Figure 7:**
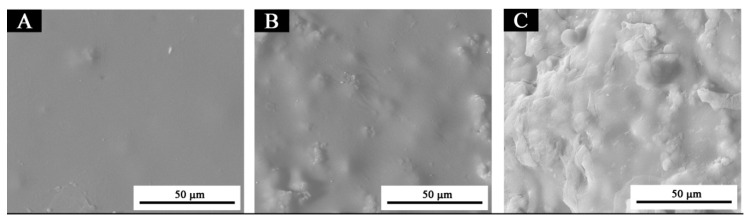
SEM image of paint film with different contents of microcapsules (the ratio of core materials to shell material in mass of 0.65). (**A**) 1.0% microcapsule addition, (**B**) 4.0% microcapsule addition, (**C**) 20.0% microcapsule addition.

**Figure 8:**
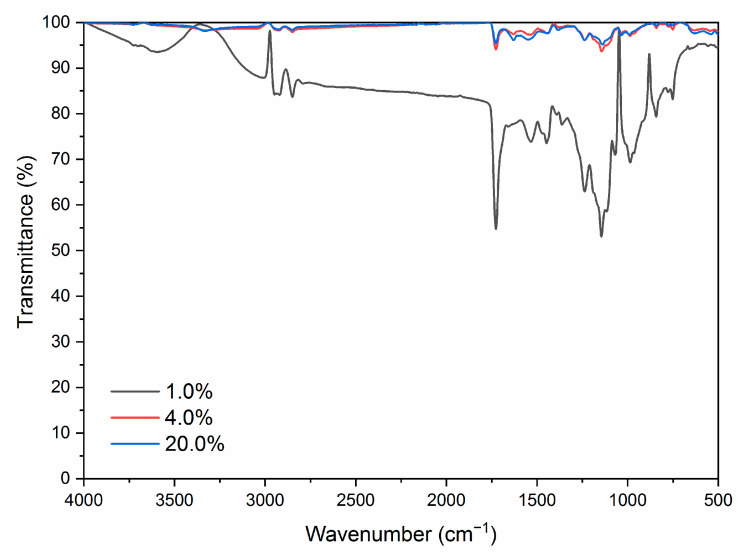
Infrared spectrum image of paint film with different contents of microcapsules (the ratio of core materials to shell material in mass of 0.65).

According to the results of optical and mechanical performances analysis, the paint film with 7.0% microcapsules has good optical and mechanical performances. The distribution of microcapsules with the ratio of core materials to shell material in mass of 0.65 in coating film was analyzed by SEM to determine the distribution of microcapsules in coatings. From Figure 7, with the increasing amount of microcapsules, the articulate sensation in the coatings on the Basswood surface becomes more obvious, which is not conducive to the uniform dispersion of coatings. The agglomeration of microcapsules in the coating becomes obvious, and the coating surface becomes more rough. Therefore, the higher the amount of microcapsules added, the more particles in the SEM, the greater the impact on the waterborne coating on the wood surface, and the more unfavorable it is for the paint film to play its own decoration and modification role. Three coatings with different microcapsule contents were tested by infrared spectroscopy, as shown in Figure 8. Compared with the waterborne coating with 1.0% microcapsule addition, the peaks of infrared spectral characteristic absorption peaks of the waterborne coating with 4.0% and 20.0% microcapsule addition decreased but did not disappear.

### 3.4. Aging Resistance Test

**Figure 9:**
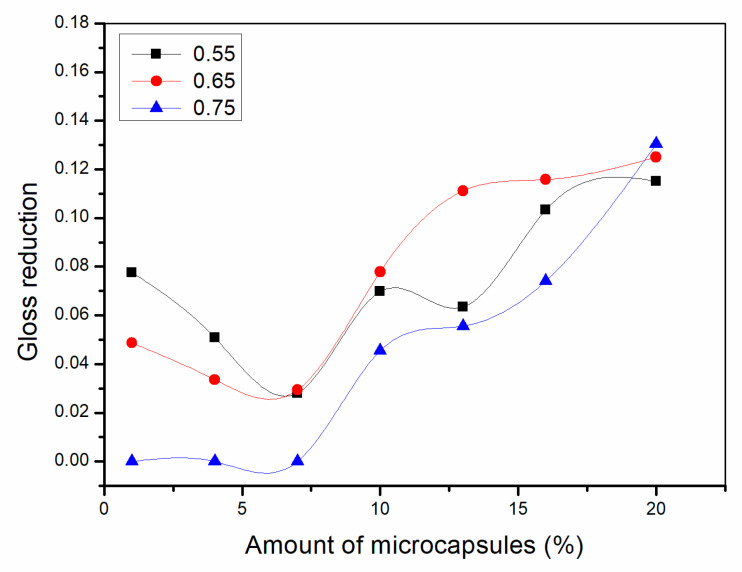
Gloss reduction in the coating after drying at 120 °C for 2 h.

**Figure 10:**
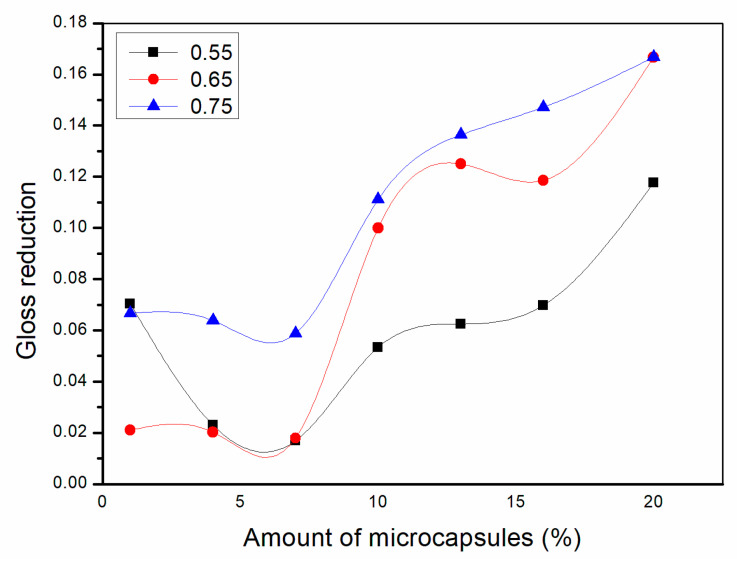
Gloss reduction in the coating after drying at 140 °C for 2 h.

**Figure 11:**
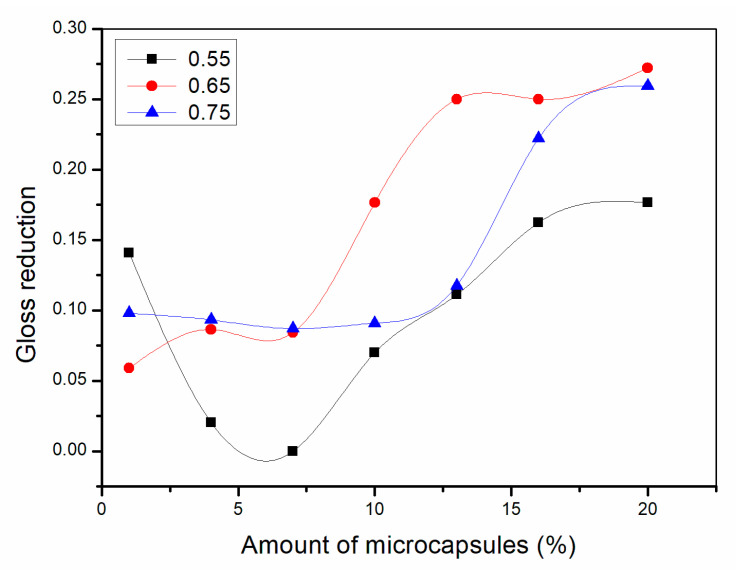
Gloss reduction the coating after drying at 160 °C for 2 h.

**Figure 12:**
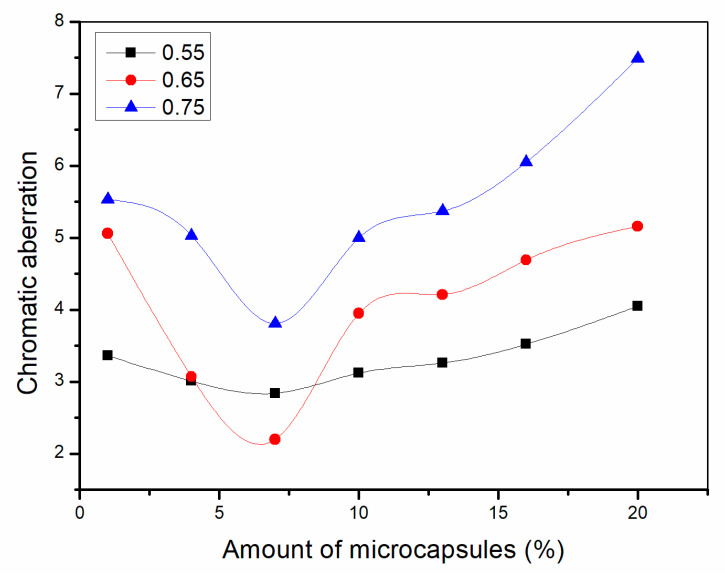
Chromatic aberration of the coating after drying at 120 °C for 2 h.

**Figure 13:**
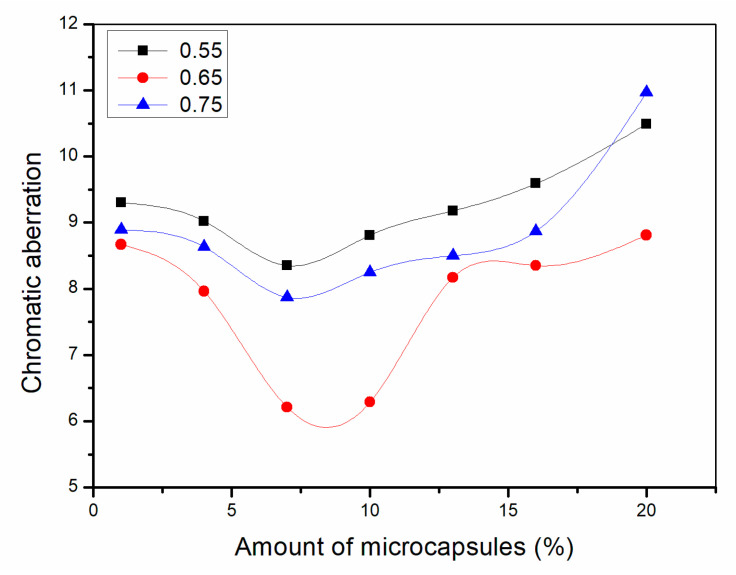
Chromatic aberration of the coating after drying at 140 °C for 2 h.

**Figure 14:**
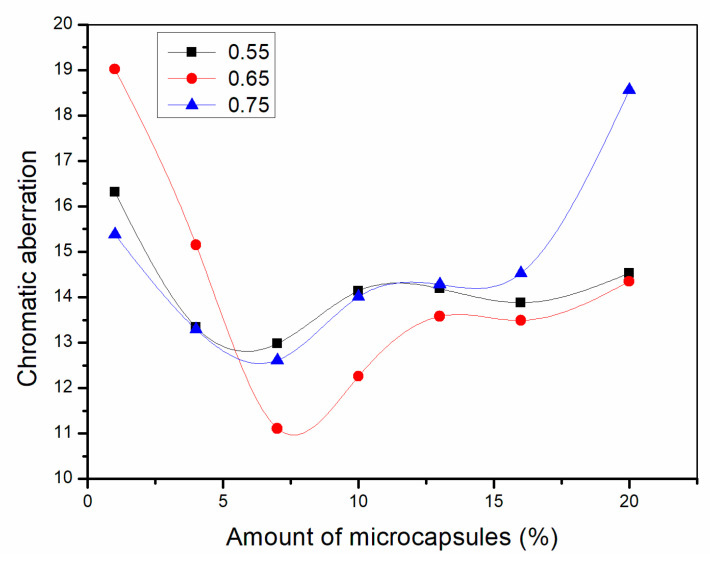
Chromatic aberration of the coating after drying at 160 °C for 2 h.

A high temperature aging test and UV aging test were carried out on waterborne coatings on Basswood surface to simulate adverse conditions of coatings in daily use. The aging resistance of the coating film can be judged according to the change of gloss and chromatic aberration before and after aging. If the aging resistance of the coating is better, the coating will keep its original gloss and color all the time. The gloss decreases slowly and the chromatic aberration is small. From Figure 9, Figure 10, Figure 11, Figure 12, Figure 13 and Figure 14, the general trend is that the higher the aging temperature of the paint film, the greater the gloss reduction and chromatic aberration of the waterborne coating on the Basswood surface. After being aged at high temperature, the gloss reduction and chromatic aberration showed a trend of decreasing first and then there is a positive correlation with the amount of microcapsules. When the amount of microcapsules is between 4.0% and 10.0%, it is the lowest. The reason may be agglomeration when the microcapsule content is too high, which leads to the decrease in aging resistance. The aging resistance of the coating is the most significant when the microcapsule content is 4.0–10.0%. In addition, when the ratio of core materials to shell material in mass is 0.65 and the addition amount is 4.0–10.0%, the chromatic aberration at different temperatures is the most stable and the value is small, so the aging resistance of the coating under this condition is better. According to the results of optical and mechanical performances, the waterborne coatings on Basswood surface had the best comprehensive performance when the ratio of core materials to shell material in mass of microcapsules was 0.65 and the content was 7.0%.

The aging resistance test results of the coatings without and with microcapsules when the ratio of core materials to shell material in mass of microcapsules was 0.65 and the content was 7.0% are compared, as shown in Table 5. The gloss reduction in the coatings without microcapsules after high temperature heating and UV aging is very high, which indicates that there is a significant decline in the performance during the aging process. The gloss value of the coating added with microcapsules decreased by only 1.3 after aging test, and the chromatic aberration value of the coating was also smaller than the other one. After the microcapsules are broken, the released repairing agent repairs the microcracks of the coating, which improves the aging resistance of the coating to maintain the initial properties of coatings. The aging resistance of coating is obviously improved after adding the microcapsules. In the actual use of wooden furniture, the surface gloss of the coating can be maintained for a long time and the service life of the furniture can be prolonged.

### 3.5. Self-Healing Effect Test

**Figure 15:**
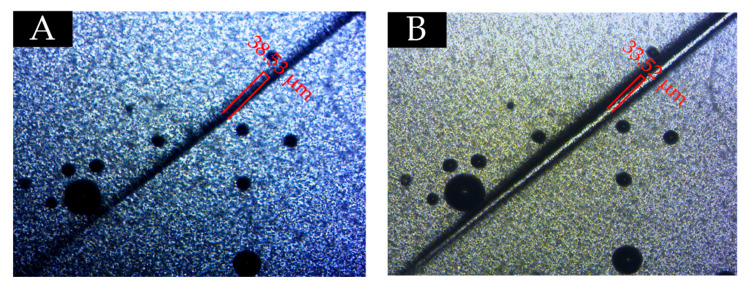
Scratch width of the coating without microcapsules: (**A**) scratch image and (**B**) scratch image after 48 h.

**Figure 16:**
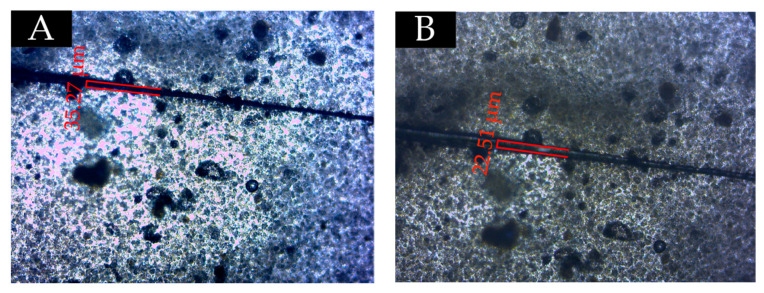
Scratch width of the coating with microcapsules: (**A**) scratch image and (**B**) scratch image after 48 h.

From the above analysis, at the content of 7.0%, the microcapsules with the ratio of core materials to shell material in mass of 0.65 can give the coating less chromatic aberration, excellent gloss, uniform surface color, better mechanical properties, and good aging resistance. Therefore, in the self-healing experiment, the effect of 0.65 the ratio of core materials to shell material in mass of microcapsules on the repairing performance of coatings at a content of 7.0% was studied. Scratches were made on the surface of waterborne coatings with microcapsules and without microcapsules. As shown in Figure 15A and Figure 16A, scratches were observed under an optical microscope. After standing at room temperature for 48 h, the scratches were observed again under the microscope. From Figure 15B and Figure 16B, the scratch on the coating without microcapsules has no self-healing effect, while the scratch on the coating with microcapsules has the self-healing phenomenon, and the scratch width has been reduced. Therefore, it is significant that the fluororesin as a healing agent is released from the cracked microcapsule to repair the scratches at room temperature when scratches appear on the surface of coatings. It has been proved that fluororesin microcapsules are effective in the self-healing process of scratches on the surface of waterborne coating.

## 4. Conclusions

With the increase in microcapsule content and the ratio of core materials to shell material in mass, the gloss of coatings decreases, the chromatic aberration increases, and the adhesion of the coatings gradually weakens although the difference is not significant. The impact resistance of coatings first increases and then decreases with the increasing concentration of microcapsules content. Microcapsule content plays a very important role in film hardness. There is a positive correlation between film hardness and microcapsule content, and a negative correlation between film hardness and microcapsule ratio of core materials to shell material in mass. With the ratio of core materials to shell material in mass of 0.65 and the increase in the addition from 1.0% to 20.0%, the chromatic aberration of waterborne coatings increased from 0.3 to 2.7, the 60° gloss decreased from 43.2% to 1.9%, the hardness increased from H to 4H, and the adhesion was basically unchanged at level 0. When the addition amount is 7.0%, the impact resistance of coatings is the best, which is 30 cm. When the addition amount is 4.0–10.0%, the waterborne coating with 0.65 mass ratio of core materials to shell material of microcapsules has the best aging resistance. Based on the above results, with 0.65 ratio of core materials to shell material of microcapsules, the comprehensive performance of Basswood surface waterborne paint film is good at a content of 7.0%. The fluororesin microcapsules have a certain repairing effect on the waterborne coatings. The results of this study provide an important technical groundwork for improving the performance of coatings on Basswood surface. The micro combination between Basswood and microcapsule coating and the durability of coating after crack repair will be further discussed.

## Figures and Tables

**Table 1:** Main components of fluororesin microcapsules.

Urea (g)	Formaldehyde (g)	PVDF (g)	Waterborne Finish (g)	NMP (g)	Mass Ratio of Core Materials to Shell Material
20.00	27.00	1.65	14.85	13.20	0.55
20.00	27.00	1.95	17.55	15.60	0.65
20.00	27.00	2.25	20.25	18.00	0.75

**Table 2:** Effect of microcapsule content on impact resistance of paint film.

Addition (%)	Impact Strength of Paint Film with the Ratio of Core Materials to Shell Material in Mass of 0.55 (cm)	Impact Strength of Paint Film with the Ratio of Core Materials to Shell Material in Mass of 0.65 (cm)	Impact Strength of Paint Film with the Ratio of Core Materials to Shell Material in Mass of 0.75 (cm)
0	10.0	10.0	10.0
1.0	25.0	25.0	15.0
4.0	20.0	15.0	20.0
7.0	20.0	30.0	20.0
10.0	25.0	25.0	20.0
13.0	30.0	15.0	20.0
16.0	20.0	15.0	20.0
20.0	15.0	20.0	5.0

**Table 3:** Effect of microcapsule content on film hardness.

Addition (%)	Film Hardness with Ratio of Core Materials to Shell Material in Mass of 0.55	Film Hardness with Ratio of Core Materials to Shell Material in Mass of 0.65	Film Hardness with Ratio of Core Materials to Shell Material in Mass of 0.75
0	H	H	H
1.0	4H	2H	HB
4.0	4H	2H	HB
7.0	4H	3H	HB
10.0	4H	3H	H
13.0	4H	3H	H
16.0	5H	3H	H
20.0	5H	4H	H

**Table 4:** Effect of microcapsule content on film adhesion.

Addition (%)	Film Adhesion with Ratio of Core Materials to Shell Material in Mass of 0.55 (Level)	Film Adhesion with Ratio of Core Materials to Shell Material in Mass of 0.65 (Level)	Film Adhesion with Ratio of Core Materials to Shell Material in Mass of 0.75 (Level)
0	0	0	0
1.0	0	0	0
4.0	0	0	0
7.0	0	0	0
10.0	0	0	1
13.0	0	0	1
16.0	0	0	1
20.0	1	0	3

**Table 5:** Effect of the microcapsules on gloss, chromatic aberration and gloss reduction after aging test.

Samples (#)	Chromatic Aberration before Aging Test	Gloss before Aging Test (%)	Chromatic Aberration after High Temperature Heating	Gloss Reduction after High Temperature Heating (%)	Chromatic Aberration after UV Aging	Gloss Reduction after UV Aging
without microcapsules	0.7	84.0	12.6	44.5	10.6	59.8
with microcapsules	0.8	5.9	11.1	0.1	7.3	1.3

## Data Availability

Not applicable.
